# High sodium food consumption pattern among Malaysian population

**DOI:** 10.1186/s41043-021-00230-5

**Published:** 2021-05-31

**Authors:** Mohamad Hasnan Ahmad, Cheong Siew Man, Fatimah Othman, Feng J. He, Ruhaya Salleh, Noor Safiza Mohammad Noor, Wan Nur Khairunnisa Wan Kozil, Graham MacGregor, Tahir Aris

**Affiliations:** 1 Institute for Public Health, National Institutes of Health, Ministry of Health Malaysia, Setia Alam, Selangor Malaysia; 2 Wolfson Institute of Preventive Medicine, Barts and The London School of Medicine and Dentistry, Queen Mary University of London, London, UK; 3 Policy and Strategic Planning Section, Allied Health Science Division, Ministry of Health Malaysia, Putrajaya, Malaysia

**Keywords:** High sodium food, Sodium consumption, Salt consumption, Population study, Malaysia, MyCoSS

## Abstract

**Background:**

Sodium is an essential mineral needed by the human body that must be obtained from food. An excess intake, however, can lead to many diseases. As food is the main source of sodium, this study aims to provide information on high sodium food consumption patterns in the Malaysian adult population.

**Methods:**

The Malaysian Community Salt Study (MyCoSS) was a nationwide cross-sectional study, conducted between October 2017 and March 2018. A multistage complex sample was applied to select a nationally representative sample of respondents aged 18 years and above. Face to face interview by a validated Food Frequency Questionnaire (FFQ) comprising 104 food items was used to gain information on high sodium food consumption patterns.

**Results:**

A total of 1047 respondents were involved in this study, with 1032 (98.6%) answering the FFQ. From the number, 54.1% exceed the recommendation of sodium intake <2000mg/day by FFQ assessment. The results also demonstrated that fried vegetables (86.4%) were the most common high sodium food consumed, followed by bread (85.9%) and omelet (80.3%). In urban areas, bread was the most common while fried vegetables took the lead in rural areas. By sex, bread was most commonly eaten by males and fried vegetables by females. The results also found that kolok mee/kampua mee contributed the highest sodium, 256.5mg/day in 9.0% adult population, followed by soy sauce 248.1mg/day in 33.2% adult population, and curry noodles 164.2mg/day in 18.5% adult population.

**Conclusion:**

Fried vegetables, bread, and soy sauce were the main source of sodium consumption among adult. Reducing the amount of sodium added to these foods should be the top priority to reduce population sodium intake and thereby prevent sodium-related diseases in Malaysia.

## Background

Sodium is an essential mineral needed by the human body that must be obtained from food. The role of sodium is to maintain electrolyte and water balance in the human body. It is also important in nerve and muscle function [1]. However, high consumption of sodium can lead to non-communicable diseases (NCDs) especially cardiovascular disease [2]. A recent study also suggests that salt or sodium intake is a potential risk factor for obesity, another major concern in public health [3].

In Malaysia, the prevalence rates of diseases associated with high sodium intake are high. For example, the overall prevalence of hypertension (known and undiagnosed) among adults of 18 years and above in Malaysia in 2015 was 30.3%. Meanwhile, the prevalence of obesity in the same age group was 17.7% [4]. There is no sign of decline in these diseases which prompts an urgent need to find comprehensive and effective prevention strategies.

Based on compelling evidence, the best strategy to help prevent diseases related to high sodium consumption is to limit dietary intake of sodium [5]. The World Health Organization (WHO) recommends a reduction to below 90 mmol (< 5 g salt) per day or < 2000 mg/day sodium in the adult general population [6]. The Recommended Nutrient Intake (RNI) 2017 for Malaysia suggests a sodium requirement of 1500 mg/day for adults 19 years and above [7].

By knowing the sodium requirement and foods with high in sodium, the individual can easily plan for their daily food intake by avoiding high-sodium foods. At the same time, the public health sector or related agencies can plan for effective intervention by understanding the pattern of high sodium food consumption in the population. Therefore, this study aims to provide baseline information on high sodium food consumption patterns in the Malaysian population.

## Methods

### Study design and study population

Data were taken from the Malaysian Community Salt Survey (MyCoSS), a nationwide cross-sectional study conducted between October 2017 and March 2018. The target population was respondents resided in the non-institutional living quarters. Those staying in institutional residents such as hotel, hostels, hospitals, and prisons were excluded from the survey. Pregnant women, those who began diuretic therapy in the last 2 weeks, having kidney disease and any condition that limit their ability to collect 24-h urine were also excluded from this study. Ethical approval was obtained from the Medical Research Ethics Committee (MREC), Ministry of Health, and Queen Mary (University of London) Research Ethics Committee, UK. Signed informed consent form was obtained from each respondent before the interview and collection of urine samples.

### Sample size

The sample size was calculated using a formula for estimating population prevalence. Sampling was designed to cover both urban and rural areas for every state in Malaysia. Calculations were done on all objectives as listed, and the biggest sample size was on the knowledge on the effect of high salt on health. We applied the findings of the previous salt study among the health workers in Malaysia (MySalt 2015) [8]. With the prevalence on knowledge on the effect of high salt on health of 6%, confidence level of 95%, estimated design effect of 1.5, the optimum sample size for a stratum was 520 respondents. The sample size was then inflated 25% to cover for non-responses; hence, the sample size for each stratum was 650 respondents, estimated sample size was 1300 respondents.

### Food frequency questionnaire (FFQ)

The Food Frequency Questionnaire (FFQ) consisting of 104 food items in 11 food groups was used to gain information on high sodium food consumption pattern. This FFQ has been used in the Malaysian Adult Nutrition Survey (MANS) 2014 with some modification and selection of high sodium food by the expert in nutrition [9]. Ten experts in nutrition applied several findings and discussed before finalizing all 104 food items to include in this FFQ. Respondents were asked in detail about each food item in a face-to-face interview by trained research assistants. Respondents answered all 104 food items in terms of frequency of intake either on a daily, weekly, or monthly basis and the number of servings they consumed during each time they ate the food. If the respondent did not consume the food on a monthly basis, they answered “0” in the per/month column.

### Data analysis

The data were analyzed using IBM SPSS version 23 for Windows (SPSS Inc., Chicago, IL, USA). Sample weight was calculated by determining the base or design weight and adjusted for study non-responses. The sampled unit weight represented the probability of it being selected into the sample. The final weight used in the analysis was the post-stratification weight referring to the existing Malaysian population information provided by DOSM. Since this study applied a complex sampling design, the analysis was conducted accordingly with 95% confidence interval. For FFQ, the conversion of food frequency to the amount of sodium in food intake was carried out using the following formula:

Amount of sodium (g) per day = frequency of intake (convert per day) × serving size × total number of servings × weight (in gram) of food in one serving × amount of sodium in 1 g of the food.

Descriptive statistics were used for describing the characteristics of the respondent and patterns of high sodium food consumption.

## Results

A total of 1047 respondents were involved in this study making a response rate of 80.5%. About 1032 respondents (98.6%) completed the FFQ. Majority respondents aged 55 to 64 years old and there were more women (59.1%) involved in this study compared to men (40.9%). More respondents were from rural areas (58.5%) compared to urban areas (41.5%) (Table 1). About 54.1% of adults were found to exceed the sodium intake recommendation of <2000 mg/day by FFQ assessment. Our study also found that fried vegetables, white/wholemeal bread and omelet were the most preferred high sodium food which was consumed by more than 80% of the Malaysian population as shown in Table 2. These top three foods remain in their rank when the analysis focused on the strata level (Table 3) and sex (Table 4). There was a difference in the urban area when white/wholemeal bread took the first position, the same as in male (Tables 3 and 4).

**Table 1:** Sociodemographic characteristics of respondent (*N* = 1032 respondents)

Characteristics	Count	Percentage (%)
Age group (years old)		
18-24	77	7.5
25-34	152	14.7
35-44	175	17.0
45-54	210	20.3
55-64	241	23.4
65 and above	177	17.2
Sex		
Men	422	40.9
Women	610	59.1
Strata		
Urban	428	41.5
Rural	604	58.5
State		
Johor	119	11.5
Kedah	104	10.1
Kelantan	75	7.3
Melaka	36	3.5
Negeri Sembilan	47	4.6
Pahang	70	6.8
Pulau Pinang	40	3.9
Perak	90	8.7
Perlis	27	2.6
Selangor	103	10.0
Terengganu	32	3.1
Sabah/Labuan	139	13.5
Sarawak	114	11.0
WP Kuala Lumpur/WP Putrajaya	36	3.5

**Table 2:** Top 10 high sodium food most consumed by Malaysian adults (*N* = 1032 respondents)

No.	Food items	Unweighted count	Estimated population	Population consumed	mg sodium (per day)
				% (95% CI)	median (IQR)
1	Fried vegetable	870	16,775,456	86.4 (83.2, 89.0)	101.8 (44.1, 264.3)
2	White/whole meal bread	823	16,674,062	85.9 (83.1, 88.2)	59.3 (29.6, 118.6)
3	Omelet	796	15,586,616	80.3 (76.5, 83.5)	71.8 (35.9, 215.3)
4	Fried chicken with spices	713	14,034,531	72.3 (67.9, 76.2)	62.5 (29.2, 125.0)
5	Fried rice	683	13,609,154	70.1 (65.8, 74.0)	95.4 (44.5, 190.8)
6	*Nasi Lemak* (*set*)	622	13,021,095	67.0 (61.7, 72.0)	88.0 (58.7, 251.5)
7	*Roti canai/roti telur*	613	12,623,157	65.0 (60.7, 69.1)	99.3 (66.2, 283.7)
8	Fried meehoon	624	11,963,462	61.6 (56.8, 66.2)	53.7 (26.9, 115.1)
9	Fried noodle	626	11,914,301	61.3 (56.0, 66.5)	82.8 (41.4, 177.4)
10	Chicken curry	583	11,143,152	57.4 (52.4, 62.2)	10.1 (4.5, 19.3)

**Table 3:** Top 10 high sodium food most consumed by Malaysian adults by strata

Food items	Urban (n=428)		Food items	Rural (n=604)	
	Population consumed, % (95% CI)	mg sodium (per day), median (IQR)		Population consumed, % (95% CI)	mg sodium (per day), median (IQR)
White/whole meal bread	88.9 (85.4, 91.6)	59.28 (29.6, 133.4)	Fried vegetable	82.8 (77.9, 86.8)	88.1 (33.0, 231.3)
Fried vegetable	87.4 (83.3, 90.6)	132.2 (44.1, 264.3)	White/whole meal bread	75.5 (71.4, 79.2)	31.1 (13.8, 88.9)
Omelet	81.7 (76.9, 85.8)	71.8 (33.5, 215.3)	Omelet	75.2 (71.2, 78.9)	98.3 (33.5, 215.3)
Fried chicken with spices	72.5 (67.0, 77.4)	62.5 (29.2, 125.0)	Fried chicken with spices	71.3 (65.6, 76.5)	62.5 (29.2, 125.0)
Fried rice	71.5 (66.2, 76.3)	95.4 (44.5, 190.8)	Fried rice	65.1 (59.1, 70.7)	95.4 (44.5, 190.8)
*Nasi Lemak* (set)	69.5 (62.9, 75.5)	88.0 (44.0, 192.8)	*Nasi Lemak* (*set*)	58.6 (51.3, 65.5)	88.0 (44.0, 251.5)
*Roti canai/roti telur*	65.8 (60.5, 70.8)	99.3 (66.2, 283.7)	*Roti canai/roti telur*	62.2 (55.5, 68.4)	99.3 (33.1, 141.9)
Fried meehoon	61.8 (55.6, 67.7)	53.7 (26.9, 115.1)	Fried meehoon	60.9 (55.9, 65.7)	53.7 (26.9, 115.1)
Fried noodle	61.7 (54.7, 68.2)	82.8 (41.4, 177.4)	Fried noodle	60.2 (55.4, 64.9)	82.8 (41.4, 177.4)
Chicken curry	57.7 (51.3, 63.7)	9.7 (4.5, 19.3)	Chicken curry	56.5 (51.1, 61.7)	9.0 (4.5, 19.3)

**Table 4:** Top 10 high sodium food most consumed by Malaysian adults by sex

Food items	Male (***n***=422)		Food items	Female (***n***=610)	
	Population consumed, % (95% CI)	mg sodium (per day), median (IQR)		Population consumed, % (95% CI)	mg sodium (per day), median (IQR)
White/whole meal bread	84.4 (80.2, 87.8)	59.3 (20.8, 118.6)	Fried vegetable	89.5 (85.9, 92.3)	117.2 (44.1, 264.3)
Fried vegetable	83.2 (77.8, 87.5)	88.1 (33.0, 231.3)	White/whole meal bread	87.3 (83.3, 90.4)	55.3 (20.8, 118.6)
Omelet	82.4 (77.1, 86.7)	100.5 (50.2, 215.3)	Omelet	78.1 (73.3, 82.3)	71.8 (33.5, 215.3)
Fried chicken with spices	76.2 (71.1, 80.7)	62.5 (29.2, 128.2)	Fried rice	68.9 (63.4, 73.9)	95.4 (44.5, 190.8)
*Roti canai/roti telur*	72.8(66.9, 78.0)	141.9 (66.2, 283.7)	Fried chicken with spices	68.3 (62.1, 74.0)	62.5 (29.2, 125.0)
Fried rice	71.2 (65.4, 76.4)	95.4 (44.5, 190.8)	*Nasi Lemak* (set)	65.2 (58.8, 71.0)	88.0 (29.3, 125.7)
*Nasi Lemak* (set)	68.9 (62.2, 75.0)	121.5 (58.7, 251.5)	Fried meehoon	64.6 (59.6, 69.3)	53.7 (26.9, 115.1)
Fried noodle	63.5 (57.0, 69.5)	124.2 (41.4, 177.4)	Fried noodle	59.2 (52.3, 65.8)	82.8 (41.4, 177.4)
Chicken curry	60.2 (54.5, 65.6)	13.5 (4.5, 20.3)	*Roti canai/roti telur*	57.2 (51.5, 62.8)	66.2 (33.1, 141.9)
Fried meehoon	58.6 (51.0, 65.7)	53.7 (26.9, 115.1)	Chicken soup	56.0 (49.7, 62.0)	36.4 (13.6, 81.6)

Mee kolok or kampua mee and light soy sauce contributed the highest sodium consumption in individuals with median consumption of 256.5 mg/day and 248.1 mg/day respectively. However, the percentage of individuals who consumed these two food items was only 9.0% and 33.2%, respectively (Table 5). Those who consumed ≥2000 mg sodium per day were found to have a higher intake of fried vegetable, *roti canai/roti telur*, omelet, fried rice, and chicken curry compared to those who consumed less than 2000 mg sodium per day according to 24-h urinary sodium analysis (Table 6).

**Table 5:** Top 10 food sources (food item) with the highest sodium consumption among Malaysian adults

No.	Food items	Unweighted count	Estimated population	Population consumed, % (95% CI)	mg sodium (per day), median (IQR)
1	Kolok mee/kampua mee	118	1,749,135	9.0 (6.2, 13.0)	256.5 (171.0, 366.4)
2	Light soy sauce	351	6,443,736	33.2 (29.0, 37.7)	248.1 (88.6, 630.4)
3	Curry noodle	155	3,584,403	18.5 (14.4, 23.3)	164.2 (82.1, 246.4)
4	Vegetable with soy sauce/oyster sauce	260	5,234,602	27.0 (23.3, 30.9)	154.9 (51.6, 387.3)
5	Fried instant noodle	186	3,754,633	19.3 (15.6, 23.8)	122.3 (61.2, 244.6)
6	Noodle soup	299	5,537,271	28.5 (24.8, 32.6)	103.5 (69.0, 206.9)
7	Vegetable cooked with salted fish	161	3,469,039	17.9 (14.1, 22.4)	103.2 (34.4, 221.2)
8	Fried vegetable	870	16,775,456	86.4 (83.2, 89.0)	101.8 (44.1, 264.3)
9	*Roti canai/roti telur*	613	12,623,157	65.0 (60.7, 69.1)	99.3 (66.2, 283.7)
10	Fried rice	683	13,609,154	70.1 (65.8, 74.0)	95.4 (44.5, 190.8)

**Table 6:** Top 10 food consumption by high sodium intake of ≥2000 mg/day sodium and less than <2000 mg/day sodium

Food items	High sodium intake (≥2000 mg/day sodium) (***n***=370)		Food items	Low sodium intake (<2000 mg/day sodium) (***n***=586)	
	% consuming (95% CI)	mg sodium (per day), median (IQR)		% Consuming (95%CI)	mg sodium (per day), median(IQR)
Fried vegetable	87.5 (83.7, 90.4)	115.7 (44.0, 246.72)	White/whole meal bread	87.3 (81.9, 91.2)	59.3 (29.6, 133.4)
White/whole meal bread	85.0 (81.2, 88.1)	59.3 (29.6, 118.6)	Fried vegetable	81.7 (75.7, 86.5)	88.6 (44.1, 264.3)
Omelet	82.5 (78.7, 85.8)	100.5 (50.2, 215.3)	Omelet	75.8 (68.2, 82.1)	71.8 (33.5, 213.3)
Fried chicken with spices	71.7 (67.3, 75.7)	62.5 (29.2, 131.3)	Fried chicken with spices	72.0 (65.2, 77.9)	62.5 (29.2, 125.0)
Fried rice	69.1 (63.1, 74.5)	95.4 (44.5,278.3)	Fried rice	71.5 (65.1, 77.1)	66.8 (33.4, 143.1)
*Nasi Lemak* (*set*)	68.4 (63.2, 73.1)	88.0 (58.7,251.5)	*Roti canai/roti telur*	67.8 (61.4, 73.6)	66.2 (33.1, 141.9)
*Roti canai/ roti telur*	65.2 (60.0, 70.0)	141.9 (66.2, 283.7)	*Nasi Lemak* (*set*)	67.2 (59.4, 74.1)	88.0 (29.3, 188.8)
Fried meehoon	61.9 (54.5, 68.9)	53.7 (26.9, 115.1)	Fried noodle	64.2 (56.8, 71.0)	82.8 (41.4, 177.4)
Fried noodle	60.0 (53.0, 66.6)	82.8 (41.4, 177.4)	Fried meehoon	61.5 (55.4, 67.3)	53.6 (26.8, 107.4)
Chicken curry	57.3 (51.4, 63.0)	13.5 (4.5, 19.3)	Chicken curry	56.5 (49.5, 63.2)	9.0 (4.5, 19.3)

## Discussion

This nationwide survey showed that the top five high sodium foods most frequently consumed by Malaysian adults were fried vegetables, white bread/wholemeal bread, omelet, fried chicken with spices and fried rice. Generally, the most popular fried vegetables in Malaysia is stir-fried like spinach [10]. The findings are almost similar to national data on food consumption statistics among Malaysians, where about 80% of the Malaysian adults reported to consume white bread/wholemeal bread [11]. However, as the study focused on high sodium food, further comparisons cannot be done with Malaysian food consumption statistics database.

Prepared or home cooked food, such as fried vegetable, omelet, fried chicken, and fried rice, are believed to contribute the highest sodium intake in Malaysia based on the high prevalence of consumption. Data from the International Study of Macro-and Micro-Nutrients (INTERMAP) study in China found that most dietary sodium came from salt added during preparing or cooking [12]. The same study reported that in developed countries, such as Japan, UK, and USA, most of the sodium came from processed foods.

The pattern of food preference was almost similar in urban or rural area and in men or women. However, in urban area and in men, white/wholemeal bread was more preferred compared to fried vegetables. A review paper from the Dublin Institute of Technology regarding food consumption trends and drivers also reported that urbanization was the main factor or drive to the high intake of processed food such as bread [13]. A cross-sectional study among 300 Malaysian university students also showed similar findings when men tend to consume more industrial processed food that was high in sodium, compared to women who mainly obtained sodium from prepared food [14].

The concern was on the consumption of *roti canai/roti telur* (flatbread) and fried noodles in men due to the high prevalence and high sodium from these foods. Rather than as a source of sodium, *roti canai* also contained high amount of fat from its main ingredient of margarine, and it was also cooked with cooking oil [15]. A cross-sectional study in Malaysia also found overweight adults consumed more *roti canai* and fried noodles compared to their normal weight counterpart [16].

Kolok mee or kampua mee and light soy sauce appeared as the food most contributed to sodium consumption. Light soy sauce contributed the main source of sodium in food, as it was used as a condiment in most food preparation, including in mee kolok or kampua mee. There was a global overview of national programs to encourage food industries to reduce sodium in their products. The document also emphasized that products contributing to salt or sodium in the diet usually include sauces, particularly soy sauce and fish sauce in many Asian countries [17]. A local survey among university students also reported that majority of the students added salt/soy sauce to food and dipped food in soy sauce mixed with chopped chilies/garlic and/or *sambal belacan* [14].

Looking into those who consumed more or equal to 2000 mg sodium per day, the preferred food is quite similar to the respondents who consumed sodium less than 2000 mg per day. However, the quantity of intake played a major role. Respondents who consumed sodium more than 2000 mg sodium per day ate large portions of fried vegetable, *roti canai/roti telur*, omelet, fried rice, and chicken curry compared to those who consumed less than 2000 mg sodium. Personal initiative to cut off the quantity or portion of high sodium food that they consumed can be the best strategy to reduce sodium intake individually. However, in a community approach, reduction in salt intake can be achieved by a gradual and sustained reduction in the amount of sodium added to foods by the food industries [18, 19].

## Conclusion

There are several aspects to highlight when describing patterns of high sodium food consumption among Malaysian adults. First, is the high sodium food choice, where almost similar food items were in the list of top ten. Second, is the method of preparation when most Malaysian adults obtained sodium from cooking or preparation of food. Third, is the amount of food consumed where it directly influence the sodium consumption. Therefore, an effective campaign targeting individual approach, community and also food industry is needed to prevent excessive sodium intake that linked to increased risk of hypertension and cardiovascular diseases.

## Data Availability

The datasets used and/or analyzed during the current study are available from the corresponding author on reasonable requests.

## Abbreviations

MyCoSS: Malaysian Community Salt Study
WHO: World Health Organization
RNI: Recommended Nutrient Intake
FFQ: Food Frequency Questionnaire
INTERMAP: International Study of Macro-and Micro-Nutrients
